# Learning Spelling From Meaning: The Role of Incomplete Contexts in Orthographic Learning

**DOI:** 10.1027/1618-3169/a000587

**Published:** 2023-09-29

**Authors:** Anezka Smejkalova, Fabienne Chetail

**Affiliations:** ^1^ Laboratory of Cognition, Language, and Development (LCLD), Université Libre de Bruxelles, Belgium

**Keywords:** novel word learning, orthographic learning, instance-based approach

## Abstract

**Abstract.** According to the instance-based approach, each novel word encounter is encoded as an episodic trace, including different aspects of word knowledge (orthography, semantics, phonology) and context. Experiencing the novel word again leads to reactivating the previous instances to support word identification. Accordingly, once a link between orthography and meaning is established through several instances of co-occurrence, presenting the novel word form enhances semantic learning even if the contexts are uninformative about the meaning (Eskenazi et al., 2018). Here, we investigated whether informative contexts enhance orthographic learning in the absence of the novel word form. Participants read pseudowords in three definition-like sentences, followed by three unrelated filler sentences (baseline condition), three uninformative sentences (orthographic condition), or three informative sentences with synonyms replacing the pseudoword (semantic condition). After reading, participants were better at spelling pseudowords exposed in the semantic than in the baseline condition and recalled more definitions of the pseudowords exposed in the orthographic than in the baseline condition. Such results indicate that both semantic and orthographic learning benefit from the contexts where the target information is absent. Overall, this supports the instance-based approach and contributes to the understanding of the interplay between orthography and semantics in contextual word learning.


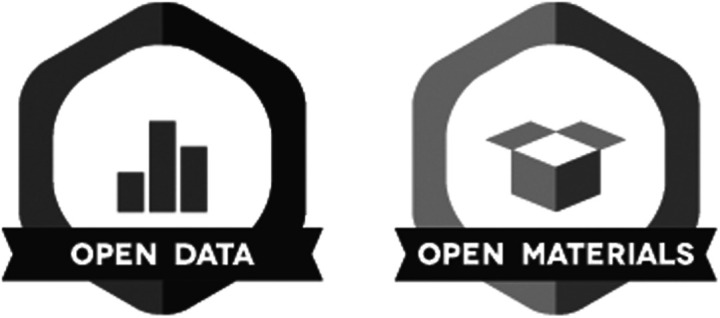


Encountering unknown words in texts represents an opportunity for word learning (e.g., Nagy & Anderson, 1984). Numerous studies highlighted that contextual encounters of a novel word lead to learning its meaning (e.g., Bolger et al., 2008; Hulme & Rodd, 2021) and spelling (e.g., Nation et al., 2007; Smejkalova & Chetail, 2023a). In context, the meaning and spelling of a novel word often co-occur, providing an occasion to create corresponding semantic and orthographic representations and link them to build a well-specified lexical entry. Such a link connecting the semantic and orthographic components is crucial because it enables the reader to easily access the meaning whenever the orthographic input is experienced again (e.g., Perfetti, 2007).

Interestingly, studies that jointly examined the outcomes of semantic and orthographic learning reported an association between meaning and spelling in recall accuracy at the item level. This suggests that producing or recognizing the correct spelling of a novel word increases the probability of recalling its definition or recognizing its meaning among several distractors (de Long & Folk, 2022; Smejkalova & Chetail, 2023a). In everyday life, readers are exposed to variable contexts when encountering new words, and the complete word-specific information, encompassing its orthography and semantics, is not always available. Indeed, some contexts are incomplete. For example, they can be uninformative (i.e., a novel word form occurs in a context that makes it difficult to infer the meaning) or informative but without the specific orthographic form (i.e., a context refers to the new concept using a synonymic or generic expression). Interestingly, Eskenazi et al. (2018) showed that after establishing a link between orthography and meaning through several instances of co-occurrence, uninformative contexts enhanced semantic learning (Eskenazi et al., 2018). However, it remains unknown whether the opposite is true, namely whether the informative contexts using a synonymic expression could enhance orthographic learning. The goal of the present study was to examine this issue to better understand the processes by which orthographic representations are built in long-term memory.

The instance-based learning approach offers a framework to account for learning information absent in a given context (Bolger et al., 2008; Reichle & Perfetti, 2003). Within this perspective, each novel word encounter is encoded as an independent trace in episodic memory. This trace contains word-specific information (e.g., spelling, semantics, syntactic role) and elements related to the actual context (e.g., words constituting the context). Experiencing the word again leads to the reactivation of existing traces through a resonance process. Activating the traces of previous encounters simultaneously with the processing of the current instance can modify this processing. Gradually, with each experience with a word contributing to its learning, the word knowledge moves from partial and context-dependent knowledge to more abstract and context-independent knowledge. This leads to the prediction that experiencing a novel word in several different contexts should foster the abstraction of its meaning through the reactivation of existing traces. Consistently, Bolger et al. (2008) found that exposing rare words in four different contexts gave rise to a better recall of abstract meanings in a definition task (i.e., participants were asked to recall the definition for each item) than exposing rare words four times in the same context. However, the difference between the two conditions was absent in the forced-choice sentence completion task (i.e., the participants had to choose among five exposed items the item that fitted in the current sentential context). In addition, the variability of the context did not reliably influence orthographic learning investigated through an orthographic choice task. The idea that the variations of the context (i.e., semantic, contextual diversity) shape the mental representation of the novel word was investigated in numerous studies (Frances et al., 2020; Hulme et al., 2023; Johns et al., 2016; Joseph & Nation, 2018; Mak et al., 2021), sometimes reporting diverging results. For example, Frances et al. (2020) showed better semantic and orthographic learning following the presentation of novel words in several different texts than several presentations in a unique text. Hulme et al. (2023) manipulated semantic diversity through the diversity of narrative scenarios. They found no effect of semantic diversity on orthographic learning. In addition, they reported that novel word meanings were better learned in nondiverse contexts (i.e., contexts involving highly similar narrative scenarios), suggesting that during the initial stages of word learning, such nondiverse contexts favor the establishment of a stable meaning representation. The role of semantic diversity was fully modelized through the semantic distinctiveness model (Jones et al., 2012), according to which the repeated contextual encounters increase the strength of novel word memory traces only if they contain contextual modulation.

However, the classical instance-based framework (Bolger et al., 2008; Reichle & Perfetti, 2003) and the hypothesis of reactivation of existing traces may still explain why complete word-specific information (i.e., orthography, semantics) can be learned from incomplete, uninformative contexts. A demonstration of that was reported by Eskenazi et al. (2018) who showed that reading uninformative sentences enhances semantic learning. They exposed participants to novel words in conditions that differed in context informativeness (informative and uninformative contexts, e.g., “She drove long distances in her fuel-efficient *snoam* last summer” for informative contexts and “The woman turned her head to look at the *snoam* just now” for uninformative contexts) and in the number of exposures. The critical comparison contrasted two conditions: a baseline condition (the participants read novel words in three informative sentences) and a mixed condition (the three informative contexts were followed by the presentation of the new word in three uninformative sentences). The participants learned novel word meanings better in the mixed condition than in the baseline, suggesting that the initial informative sentences were sufficient to create episodic traces containing orthographic and semantic information. The following novel word encounters in uninformative contexts may have reactivated and strengthened these traces. This effect was driven by the performance of high-skilled spellers, and the authors speculated that high-skilled spellers form stronger orthographic representations enabling them to efficiently learn words from incomplete contexts. However, Eskenazi et al. (2018) mostly focused on semantic learning, and it is unclear if the contexts in which the orthographic component of a word is absent may enhance orthographic learning. The orthographic information is encoded during a novel word encounter, and therefore, it could be reactivated when the semantic part is experienced again. However, accessing orthography from meaning is made difficult by the possibility of linking the semantic content to another lexical unit (e.g., synonym) through lexical selection (e.g., Alario et al., 2003; Breining & Rapp, 2019; Caramazza, 1997; Falconer & Buchwald, 2013; Levelt et al., 1999). Due to the activation of synonymous words through the selection process, a specific spelling may be less likely to be activated. Hence, it is possible that when the specific word form is absent, the informative context cannot contribute to orthographic learning.

Therefore, the goal of the present study was twofold. First, we aimed to replicate the findings reported by Eskenazi et al. (2018) regarding the role of uninformative contexts in semantic learning. Second, we sought to investigate whether informative contexts enhance orthographic learning when the novel word is absent so that we could test if the reactivation of pre-existing traces contributes to orthographic learning.

We asked the participants to read pseudowords in three definition-like sentences (the pseudowords were used here as novel words, e.g., Eskenazi et al., 2018; Smejkalova & Chetail, 2023a). These three sentences were followed by three unrelated filler sentences (baseline condition), three uninformative sentences containing the pseudowords (orthographic condition), or three sentences presenting the semantic information with synonyms replacing the pseudowords (semantic condition). Based on the instance-based framework (Bolger et al., 2008; Reichle & Perfetti, 2003; Eskenazi et al., 2018), we expected to observe uninformative contexts enhancing semantic learning. If the reactivation of episodic traces contributes to orthographic learning, we hypothesized that orthographic learning benefits from additional exposures to informative contexts, even if the specific orthographic form is absent.

## Method

### Participants

A sample of 102 participants^1^ (82 females) recruited among university students was enrolled in the study. They were adult native French speakers (18–34 years) with no history of reading-related difficulties and received 20€ for their participation.

### Materials and Design

All materials are available at https://osf.io/wgzv6/ (Smejkalova & Chetail, 2023b). The materials consisted of 96 pseudowords and 480 sentences. The pseudowords (e.g., *bicess*) were devised by changing one letter of existing French low-frequency nouns (e.g., *biceps*). The intended *correct spelling* (i.e., derived from the base word) was inconsistent (i.e., the pronunciation /*bi.sès*/ could be spelled as *bicesse*, *bissèce*, for example) and not likely to be spontaneously produced.^2^ Half of the pseudowords were embedded in exposure sentences (exposed pseudowords), and the other half served as a control condition (nonexposed pseudowords) in the spelling-to-dictation task assessing orthographic learning. We created nine sentences for each exposed pseudoword: three definition-like sentences, (i.e., pseudoword included in sentences describing its meaning with a general semantic category and specific traits), three orthography-only sentences (i.e., sentences with no cue about pseudowords meaning), and three semantic-only sentences (i.e., sentences rephrased from definition-like sentences, the target pseudoword replaced by a synonym). Table 1 shows an example of sentences for a given item. The pairing between the pseudoword and its meaning was done to avoid any obvious association driven by morphological features.^3^

**Table 1:** Examples of nine sentences related to one experimental item

	Example of sentences
Sentence type	English
Definition-like sentences	1. The *jélibat* is an exotic aquarium fish with heart-shaped pink fins.
	2. With its exotic pink heart-shaped fins, the *jélibat* is a very common aquarium fish.
	3. To get an exotic heart-shaped finned *jélibat* in the aquarium, you need to go to a pet store.
Orthography-only sentences	1. Gaia received a *jélibat* as a gift for her birthday.
	2. During play time, Gaia drew a *jélibat* on the floor with chalk.
	3. Gaia was sad because the *jélibat* drawing she had made the day before with chalk had gone with the rain.
Semantic-only sentences	1. *Exotic fish* with pink heart-shaped fins are very common aquarium fish.
	2. Gaia got a small *exotic fish* with pink heart-shaped fins for her birthday.
	3. Gaia added the small *exotic fish* with heart-shaped pink fins to her aquarium.
*Note*. The pseudowords are in italic here, but they were not highlighted on the screen. For the semantic-only sentences, the highlighted expression is a synonymic expression used instead of the target pseudoword. Note that the examples are an English translation of the original French sentences. All sentences used are at OSF in their original version.	

We used the resulting 432 sentences containing the target pseudoword and/or its definition to build three experimental conditions: baseline condition, orthographic condition, and semantic condition. The baseline condition consisted of a presentation of three definition-like sentences and three filler sentences. The orthographic condition consisted of a presentation of three definition-like sentences and three orthography-only sentences. The semantic condition consisted of a presentation of three definition-like sentences and three semantic-only sentences. Each participant was exposed to 48 pseudowords by reading 288 sentences divided into six blocks with a single sentence related to a specific pseudoword in a block. The order of sentences within each block was random. The first three blocks contained only definition-like sentences. The last three blocks contained condition-specific sentences and 48 unrelated filler sentences (16 per block). Six counterbalanced lists were created so that each exposed pseudoword occurred in each condition but in different participants. Table 2 summarizes the experimental conditions in six blocks.

**Table 2:** Summary of the experimental conditions and blocks

Blocks	Baseline condition	Orthographic condition	Semantic condition
Block 1–Block 3	For all conditions: sentences with orthographic and semantic information		
Block 4–Block 6	Filler sentences (orthographic information absent, semantic information absent)	Uninformative sentences (orthographic information present, semantic information absent)	Informative sentence (orthographic information absent, semantic information present)

### Procedure

The experiment involved two sessions on two consecutive days. On the first day, the participants completed a short demographic form and the BOQS test (Chetail et al., 2019), assessing their knowledge of French orthography through a standardized paper and pencil spelling-to-dictation task (25 items). Next, they had to read sentences with embedded pseudowords. The sentences were presented one by one on the computer screen. The participants were instructed to read sentences to understand well and press the spacebar to move from one screen to another. There was no explicit instruction concerning the pseudowords.

**Figure 1:**
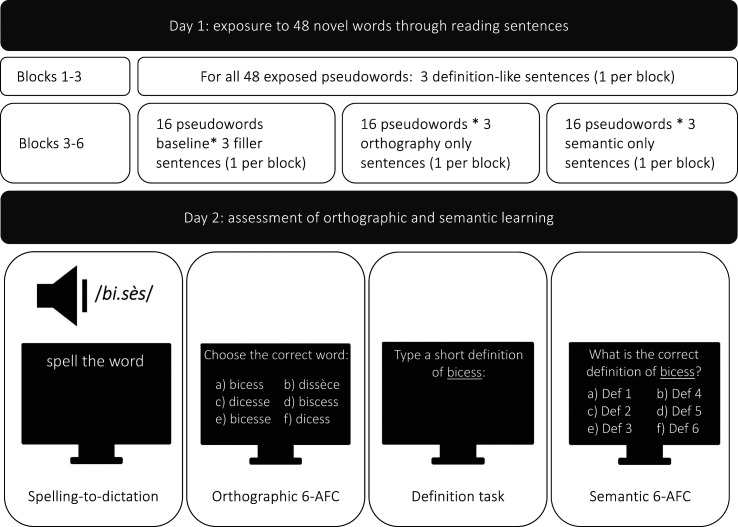
Summary of the procedure. The tasks assessing orthographic and semantic learning were administrated following the order presented in the diagram (from left to right).

On the second day, they completed the tasks assessing the novel word learning. The orthographic learning was tested with a spelling-to-dictation task and a six-alternative forced-choice task (6-AFC) and the semantic learning with a definition task and a semantic 6-AFC. In the following lines, the tasks are presented in order of administration. In the spelling-to-dictation task, participants listened to the exposed and nonexposed pseudowords and had to type them. The pseudowords were presented one by one, and the participants could replay the audio file. In the orthographic 6-AFC, they had to choose the correct orthographic form among five foils that were visually or phonologically similar (e.g., *bicess, bicesse, biscess, dicesse, dicess, dissèce*). In the definition task, the participants had to type a brief definition for each exposed pseudoword presented in isolation, with no contextual aid. In the semantic 6-AFC task, they had to select one definition among six, for each exposed pseudoword. The five semantic foils were other exposed items and new items, and each definition appeared six times (once as a target, five times as a foil). Figure 1 presents a summary of the procedure.

## Results

**Figure 2:**
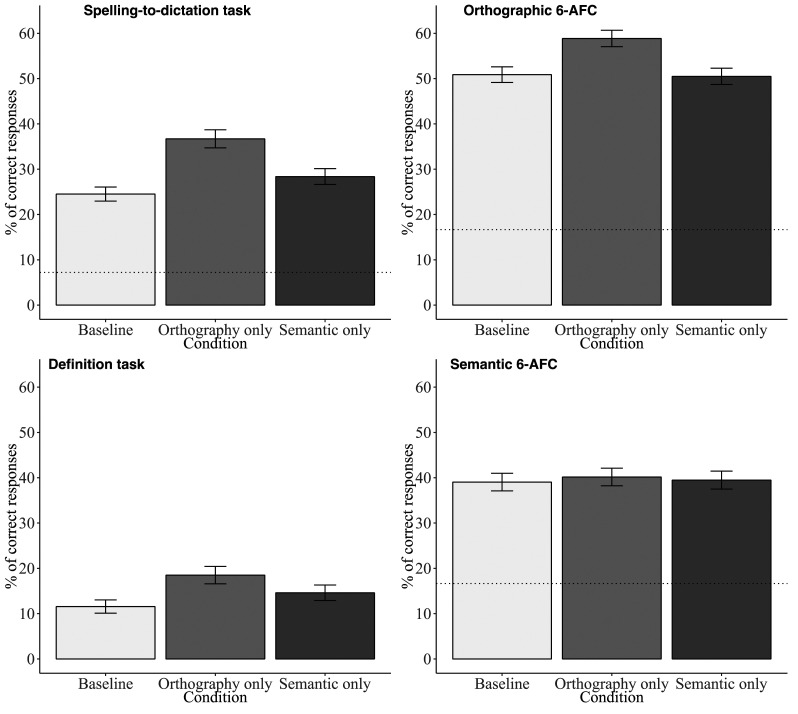
Summary of results obtained in tasks assessing orthographic and semantic learning. The error bars represent standard errors. In the spelling-to-dictation task, the dotted line represents the mean percentage of correct responses for nonexposed pseudowords. In orthographic and semantic 6-AFC, the dotted line represents the chance-level (100/6).

The analyses were run in R in the RStudio environment (RStudio Team, 2020). We computed generalized linear mixed-effect models with the lme4 (Bates et al., 2015) and the lmerTest (Kuznetsova et al., 2017) packages. The models included the learning condition as the fixed effect and the maximal random structure given the design. They were simplified when overfitting by removing the random term associated with the smallest part of the variance. For the spelling-to-dictation task, an additional model with exposure (exposed, nonexposed pseudowords) as fixed factor was estimated.^4^ To code the fixed effects, we used a treatment contrast coding to directly compare orthographic and semantic conditions to the baseline condition (see Brehm & Alday, 2022, for a detailed explanation of main contrast coding schemes used in mixed models). The raw data and scripts are available at https://osf.io/wgzv6/. Figure 2 illustrates the results obtained in each task used to assess orthographic and semantic learning.

### Orthographic Learning

Table 3 reports the mean percentage of correct responses in tasks assessing orthographic learning. In the spelling-to-dictation task, a response was considered as correct if it corresponded to the intended spelling. The participants spelled more accurately exposed (*M* = 30%) than nonexposed (*M* = 7%) pseudowords, *z* = 9.30, *p* < .001, odds ratio = 9.07. They also spelled more accurately the pseudowords exposed in orthographic condition (*M* = 37%) and in semantic condition (*M* = 28%) than in baseline condition (*M* = 25%), *z* = 7.06, *p* < .001, odds ratio = 2.06, and *z* = 2.20, *p* = .028, odds ratio = 1.27, respectively. In the orthographic 6-AFC task, the participants performed better in the orthographic condition (*M* = 59%) than in the baseline condition (*M* = 51%), *z* = 5.05, *p* < .001, odds ratio = 1.47. The difference between the semantic condition (*M* = 51%) and the baseline (*M* = 51%) was not significant, *z* = −0.22, *p* = .830, odds ratio = 0.98.

**Table 3:** Descriptive statistics (means, standard deviations [*SD*], minimal and maximal values) of correct responses (in %) in the tasks assessing orthographic and semantic learning

Tasks	*M*	*SD*	Min–Max
Spelling-to-dictation			
Nonexposed	7	5	0–25
Exposed	30	16	2–79
Baseline	25	16	0–81
Orthographic	37	20	0–81
Semantic	28	18	0–88
Orthographic 6-AFC			
Baseline	51	17	6–100
Orthographic	59	18	19–100
Semantic	51	18	13–94
Definition			
Baseline	12	15	0–75
Orthographic	19	19	0–81
Semantic	15	17	0–75
Semantic 6-AFC			
Baseline	39	20	6–100
Orthographic	40	20	6–100
Semantic	39	20	6–94

### Semantic Learning

Table 3 reports the mean percentage of correct responses in both tasks assessing semantic learning. In the definition task, the response was counted as correct if the precise definition (e.g., *vehicle using solar energy*), category (e.g., *vehicle*), or a clearly synonymic definition (e.g., *sort of car*) was given. The participants recalled better the meanings associated with pseudowords in the orthographic condition (*M* = 19%) than in the baseline condition (*M* = 12%), *z* = 6.66, *p* < .001, odds ratio = 2.20. They also recalled better the meanings associated with pseudowords in the semantic condition (*M* = 15%) than in the baseline condition, *z* = 3.03, *p* = .002, odds ratio = 1.44. In the semantic 6-AFC task, the participants performed equally well across learning conditions, the performance in the baseline condition (*M* = 39%) did not significantly differ from the orthographic condition (*M* = 40%), *z* = 0.73, *p* = .468, odds ratio = 1.06, and from the semantic condition (*M* = 39%), *z* = 0.29, *p* = .775, odds ratio = 1.02.

### The Role of the Spelling Skills: Exploratory Analysis

The mean score in the BOQS test was 62% (*SD* = 21%, range: 20–100%, skew = −0.23, kurtosis = −0.94), which is higher than the mean score reported in the normative data (*M* = 50%, Chetail et al., 2019), *t*(101) = 5.73, *p* < .001. We conducted an exploratory analysis to investigate whether spelling skills represent an advantage for learning words from incomplete contexts as suggested by Eskenazi et al. (2018). We added the BOQS as a fixed factor in all models reported above (i.e., the fixed-effect structure of the models was the following: score ∼ condition * spelling skills). The results showed an overall positive association between the spelling skills and word learning in each task (spelling-to-dictation task: *z* = 2.87, *p* = .004, odds ratio = 4.00, orthographic 6-AFC: *z* = 2.97, *p* = .003, odds ratio = 3.20, definition task: *z* = 2.10, *p* = .036, odds ratio = 6.34, semantic 6-AFC: *z* = 3.15, *p* = .002, odds ratio = 4.19). However, the spelling skills did not interact with exposure condition in any task (spelling-to-dictation task: *p* = .211, orthographic 6-AFC: *p* = .615, definition task: *p* = .683, semantic 6-AFC: *p* = .207).

## Discussion

In the present study, we aimed to investigate whether informative contexts enhance orthographic learning when the word form is absent in the context. We also aimed to replicate the findings reported by Eskenazi et al. (2018) about the role of uninformative contexts in semantic learning. The participants read pseudowords in three definition-like sentences. These three sentences were followed by three filler sentences (baseline condition), three uninformative sentences (orthographic condition), or three informative sentences with synonyms replacing the intended word form (semantic condition). We hypothesized that the orthographic condition would foster semantic learning (Eskenazi et al., 2018). Crucially, we also expected that the semantic condition would enhance orthographic learning, in line with the instance-based approach (Bolger et al., 2008; Reichle & Perfetti, 2003). Overall, the levels of orthographic and semantic learning were rather modest but within the range of values obtained in experiments that used reading words or texts to expose participants to novel words without direct instruction to memorize these novel words (e.g., Chaves et al., 2020; Pellicer-Sánchez & Schmitt, 2010; Smejkalova & Chetail, 2023a).

### Orthographic Learning

In the spelling-to-dictation task, the participants spelled better pseudowords exposed in the semantic than in the baseline condition, demonstrating that informative contexts support orthographic learning even if the word form is absent. This observation suggests that the resonance process hypothesized by the instance-based approach contributes not only to semantic learning but also to orthographic learning. Interestingly, the difference between the semantic and baseline conditions was absent in the orthographic 6-AFC task. The main difference between the spelling-to-dictation and the 6-AFC task is that the former is based on recall and the latter on recognition. Within the instance-based framework, Reichle and Perfetti (2003) distinguish between the familiarity and accessibility of memory traces. The former, related to how well the word is established in memory, is captured by recognition tasks. The latter, informing about how well the features of a given word can be retrieved, is linked to the performance in recall tasks. The discrepancy between the results observed in the two tasks could thus indicate that the resonance process preferentially affects the accessibility of memory traces.

In both orthographic tasks, the orthographic condition led to better performance than the baseline condition. This observation is consistent with previous studies highlighting the importance of the frequency effect in orthographic learning (e.g., Adlof et al., 2016; Pellicer-Sánchez & Schmitt, 2010) and with a classical interpretation of the frequency effect in word recognition as a learning effect (see Brysbaert et al., 2018 for a summary of theories of frequency effect). Surprisingly, Eskenazi et al. (2018) reported that participants recognized equally well pseudowords in the baseline condition (equivalent to our baseline condition) and in the mixed condition (equivalent to our orthographic condition). Such inconsistency suggests that the number of occurrences cannot fully explain the difference in orthographic learning efficiency between the orthographic and baseline conditions, and the contribution of other factors, such as contextual diversity (e.g., Adelman et al., 2006), should be considered to understand the present results. Indeed, contextual diversity is thought to be a better predictor of word recognition than lexical frequency (e.g., Adelman et al., 2006; Jones et al., 2012), and several studies reported that it also influenced word learning (e.g., Frances et al., 2020; Rosa et al., 2022). However, it is unclear how the material used in the present study differs in terms of contextual diversity from the material used by Eskenazi et al. (2018). This calls for studies directly addressing frequency effects in orthographic learning and controlling for the diversity of contexts used to establish the minimal contrast leading to a reliable frequency effect.

### Semantic Learning

Concerning semantic learning, the pattern of results observed in the definition task was similar to Eskenazi et al. (2018). The participants recalled more meanings of the pseudowords exposed in the orthographic than in the baseline condition. This means that uninformative contexts reinforced semantic learning. As in the orthographic learning tasks, the effect was present in the recall-based task (definition task) but absent in the recognition-based task (semantic 6-AFC). This differs from the results reported by Eskenazi et al. (2018), since they found the pattern described above with a recognition task. This discrepancy can be explained by the use of different semantic distractors. Indeed, Eskenazi et al. (2018) used only exposed items as distractors to diminish the reliance on familiarity to guess the correct answer. In the present study, we also included unexposed items as distractors. Thus, it is possible that the recognition tasks in the two studies targeted slightly different processes. Overall, the fact that the pattern was absent in the semantic 6-AFC and present in the definition task here reinforces our interpretation, according to which the resonance processes at play during the reactivation of traces preferentially affects the accessibility of memory traces.

It is worth noting that in the definition task, the participants performed better for pseudowords exposed in the semantic than in the baseline condition. This result indicates that the informative contexts, with the target orthographic form replaced by a synonym, were successfully associated with initial instances. Interestingly, the participants also performed better in orthographic than in semantic condition. One possible explanation is that the orthographic condition would benefit semantic learning more than the semantic condition because, in such cases, the semantic information could only be evoked through retrieval practice. Indeed, a retrieval opportunity leading to better learning than a novel learning episode is a classic result of memory research (see Roediger & Butler, 2011 for a review). However, following this rationale, we would also expect to find better orthographic learning in the semantic than orthographic condition, which was not the case. Alternatively, we believe that how semantic knowledge is assessed could explain this result. In the definition task, the participants had to recall the definition of a novel word based on the presentation of its orthographic form. Strictly speaking, such a task evaluates the ability to use the orthographic cues to access the word meaning, not semantic learning itself. Moreover, one of the frequent errors consisted in providing an incorrect definition that would represent a correct answer for another trial. Put differently, the participants memorized more meanings than they recalled correctly based on orthographic cues. According to us, tasks assessing semantic learning with an orthographic cue could thus lead to overestimating the effect of orthography on semantic learning. It is also interesting to point out that this overestimation could explain, at least partly, the association between semantic and orthographic learning observed in word-learning studies at the item-level (de Long & Folk, 2022; Eskenazi et al., 2018; Smejkalova & Chetail, 2023a). This observation calls for the development of novel tasks that better assess semantic learning so that we could understand how orthography interacts with meaning acquisition without such task-dependent confounds. One possible strategy could rely on balancing the cues difficulty by using both semantic and orthographic cues to test orthographic and semantic learning in recognition-based tasks.

### The Role of Spelling Skills

In present study, spelling skills contributed to the overall level of novel word learning and did not interact with exposure conditions. On the contrary, Eskenazi et al. (2018) reported that a better meaning learning from uninformative contexts was specific to high-skilled spellers. Here, to assess spelling skills, we used a French standardized orthographic quality scale (BOQS, Chetail et al., 2019). When compared with the norms of the test, the results obtained in the current sample indicates that the participants were mainly above-average spellers. We can thus assume that their spelling skills were close to those of the high-spellers in the study by Eskenazi et al. (2018) and thus sufficient to lead to significant effects. However, it is also possible that spelling skills do not reliably modulate word learning depending on context characteristics (e.g., Rosa et al., 2022). In any case, the fact that some effects may be present or absent, depending on the participant's skills, highlights the need for monitoring at least spelling skills in studies addressing novel word learning.

### Conclusion

In conclusion, the present study showed that once a link between orthography and meaning has been established through several instances of co-occurrence, subsequent contexts providing incomplete information about a novel word reinforce the learning of the absent element. Crucially, we replicated the core results of Eskenazi et al. (2018), according to which uninformative contexts support semantic learning. More importantly, we extended such a result to orthographic learning by showing that semantic knowledge supports orthographic learning from context. However, the recall and recognition-based tasks displayed a different pattern of results: The incomplete contexts enhanced orthographic and semantic learning but only in the recall-based spelling-to-dictation and the definition tasks. Such a result suggests that that instance-based mechanism preferentially affects the accessibility of memory traces. Overall, the results highlight that the predictions based on an instance-based approach are valuable to word-learning investigations based on individual experiences with a word through contextual encounters. They also contribute to the understanding of the complex interplay between orthography and semantics in contextual word learning.

## Footnotes

^1^
Sample size was defined a priori to get at least 1,600 observations per condition (Brysbaert & Stevens, 2018).

^2^
Another sample of 27 students took part in a pretest with a pseudoword spelling-to-dictation task. For the items selected in the study, the correct spelling was given by less than 15% of the participants.

^3^
Another sample of 35 students completed an association pretest. We avoided pseudoword-category pairings selected by more than 11% of participants in this pretest.

^4^
Including nonexposed pseudowords as a control condition in the spelling-to-dictation was done to verify whether orthographic learning, as measured with the spelling task, occurred independently of learning condition. The exposure and condition factors were modeled separately because the condition factor was not meaningful for nonexposed pseudowords.

## Notes

**Publication Ethics:** The study was approved by the institutional committee and complied with the ethical guidelines of the 1975 Declaration of Helsinki regarding experimentation on human subjects.

**Authorship:** Anezka Smejkalova: conceptualization, data curation, formal analysis, investigation, methods, project administration, resources, software, validation, writing – original draft preparation, writing – review & editing. Fabienne Chetail: conceptualization, supervision, validation, writing – review & editing.

**Open Data:** The information needed to reproduce all of the reported results and other supplementary materials are available at https://osf.io/wgzv6/ (Smejkalova & Chetail, 2023b).
